# Extent of Night Warming and Spatially Heterogeneous Cloudiness Differentiate Temporal Trend of Greenness in Mountainous Tropics in the New Century

**DOI:** 10.1038/srep41256

**Published:** 2017-01-25

**Authors:** Mei Yu, Qiong Gao, Chunxiao Gao, Chao Wang

**Affiliations:** 1 Department of Environmental Sciences, University of Puerto Rico, Rio Piedras, San Juan, PR 00936, USA; 2 Duke University, Durham, NC 27705, USA

## Abstract

Tropical forests have essential functions in global C dynamics but vulnerable to changes in land cover land use (LCLUC) and climate. The tropics of Caribbean are experiencing warming and drying climate and diverse LCLUC. However, large-scale studies to detect long-term trends of C and mechanisms behind are still rare. Using MODIS Enhanced Vegetation Index (EVI), we investigated greenness trend in the Greater Antilles Caribbean during 2000–2015, and analyzed trend of vegetation patches without LCLUC to give prominence to climate impacts. We hypothesized that night warming and heavy cloudiness would reduce EVI in this mountainous tropical region. Over the 15 years, EVI decreased significantly in Jamaica, Haiti, Dominican Republic, and Puerto Rico, but increased in Cuba partly due to its strong reforestation. Haiti had the largest decreasing trend because of continuous deforestation for charcoals. After LCLUC was excluded, EVI trend still varied greatly, decreasing in the windward but increasing in the leeward of Puerto Rico. Nighttime warming reinforced by spatially heterogeneous cloudiness was found to significantly and negatively correlate with EVI trend, and explained the spatial pattern of the latter. Although cooled daytime and increased rainfall might enhance EVI, nighttime warming dominated the climate impacts and differentiated the EVI trend.

Terrestrial Ecosystems shape and are shaped by regional and global changes in climate^1^,^2^. Tropical forests, accounting for over 2/3 of terrestrial live plant biomass^3^, have the largest plant carbon pool and the highest net primary productivity (NPP)^4^ among terrestrial ecosystems. Therefore, the responses and feedbacks of tropical forests to climate change are critical components of global interactions between terrestrial ecosystems and climate.

Climate changes could alter functions of tropical forests in global C cycles and weaken their sink capacity. The IPCC Assessment Report 5 documented globally-averaged warming of 0.85 °C (0.65–1.06) in 1880–2012, and predicted a likely warming of greater than 1.5 °C relative to 1850–1900 at the end of the 21^st^ century based on the major RCP (representative concentration pathways) scenarios^5^. A review of drought under global warming revealed a substantial increase of global aridity since the 1970s, and climate models predicted increased aridity over most of the Americas in the 21^st^ century^6^. Both warming and drought would reduce C sequestration capability of tropical forests and likely change them from a C sink to a C source^7^,^8^,^9^. Nighttime warming is reported to be faster than daytime in tropics and globally^5^,^10^, and the asymmetric warming affects photosynthesis and respiration differentially^10^,^11^. Tropical nighttime warming was shown to dominate the variability of terrestrial carbon sink^10^. Tropical drought and consequent C loss were also highlighted for Amazon and Congo forests^12^,^13^.

As the second largest source for carbon emission^5^, Land Cover and Land Use Change (LCLUC) is significant in the tropics, such as the deforestation in Amazon and Southeast Asia^14^,^15^. On the other hand, economy globalization, intensified agriculture, and economy shift to industry and service led to agriculture abandonment and subsequent forest regrowth in both developed and developing countries^16^,^17^. The C loss due to deforestation is compensated by the C gain from reforestation. LCLUC interacts with climate change to complicate quantification of C budget of tropical forests. It is crucial to investigate the synergetic impacts of climate change and LCLUC (deforestation and reforestation) in order to determine the C sequestration capability of tropical forests.

As an important forest region and a global biodiversity hotspot with high endemism^18^, the tropics of Caribbean are experiencing warming and drying climate^19^ and diverse LCLUC^20^. The climate in Caribbean is projected to have more frequent dry days and temperature elevation faster than global average^21^,^22^. Recent severe drought during 2014–2015 threatened the tropical ecosystems, and severely reduced water discharges in major watersheds in Puerto Rico (http://pr.water.usgs.gov/). The drought significantly impacted ecosystems functions, services, and society needs. LCLUC in the Caribbean is quite diverse^20^. For example, Puerto Rico underwent severe deforestation from the full to less than 6% forest cover during the Spain era, and then the reforestation to over 40% in the price of agriculture shrinkage to 3% after becoming the US territory^16^,^23^. In Cuba, sugarcane cultivation dropped abruptly in 1990s due to the end of subsidy from the former Soviet Union, which triggered significant reforestation. With less than $1,000 per capita GDP, Haiti still has great demand for charcoals as the main energy source, which incurs continuous deforestation. Forest cover in Haiti is the lowest (<5%, FAOSTAT, faostat3.fao.org) among the big islands of the Caribbean. Diverse LCLUC, together with dense populations and economic shift between agriculture and industry, makes the tropical ecosystem in the Caribbean vulnerable to the climate change and also amplifies the uncertainties in projection of the future C sink capabilities.

Spatiotemporal trend of greenness monitored by remote sensing reflects the impacts of both climate changes and LCLUC on carbon input and vegetation growth. Thus monitoring and understanding the trend of greenness can help quantify NPP and biomass at large scales^24^. The Enhanced Vegetation Index (EVI) dataset derived from the Moderate Resolution Imaging Spectroradiometer (MODIS), which provides global greenness coverage at the spatial resolution of 250 m for every 16 days since 2000, is widely used to detect LCLUC^25^,^26^,^27^ and to assess vegetation response to climate changes across spatiotemporal scales^13^,^28^,^29^,^30^. However, the relative importance of climate change versus LCLUC as well as the interaction between the two drivers in vegetation greenness is rarely explicitly addressed in the literature, which weakens the conclusion of climate change impact on vegetation greenness, especially for the tropical regions with diverse LCLUC.

Large-scale study of vegetation response to climate is rare in the tropics of Caribbean despite their importance in global carbon cycles and biodiversity and their vulnerability to the ongoing severe changes in climate and land use. Our objectives are: (1) to assess the temporal trend of greenness for the countries in the tropics of the Caribbean since 2000; and (2) to investigate how asymmetric warming between day and night as well as changes in rainfall and solar radiation affects the greenness over the new century. We hypothesize that: (1) Trend of greenness varies among the Caribbean countries due to diverse LCLUC; (2) Spatial patterns of asymmetric warming between day and night and cloudiness contribute to differentiate the temporal trend of greenness; and (3) Rainfall increases greenness if moisture-limited, however, cloudiness associated with rainfall might complicate the rainfall effect if radiation-limited.

## Methods

### Study area

**Figure 1:**
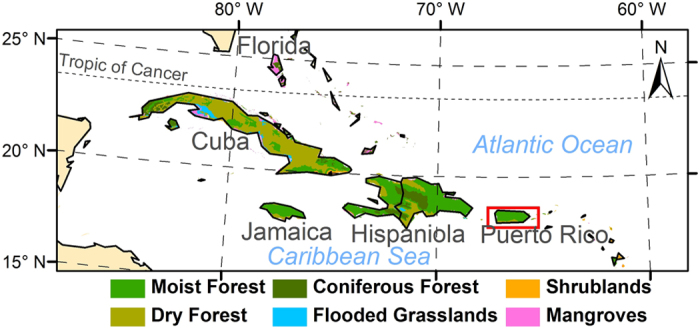
Biomes of the Greater Antilles in the Caribbean (biome data from Olson *et al*. 2001). Map created using ArcGIS 10.0 (Esri, CA, www.esri.com).

The tropic islands of Caribbean lie between the Atlantic Ocean and the Caribbean Sea. The Greater Antilles and the Lesser Antilles form an arc with the former in the north and the latter in the east. The Greater Antilles (within 65°–85°W and 17.5°–23.5°N, Fig. 1) consist of four big islands, hosting Cuba, Haiti, Dominican Republic (sharing the island of Hispaniola with Haiti), Puerto Rico, and Jamaica. Among them, Cuba is the largest (110,860 km^2^) while Puerto Rico is the smallest (8,950 km^2^) (Table 1). The prevailing trade wind comes from the northeast. Tropical moist forest and tropical dry forest are dominant biomes in the Greater Antilles^31^. Specifically, tropical moist forests dominate Puerto Rico, Jamaica, and Hispaniola, whereas tropical dry forests prevail in Cuba due to its higher latitude than the other three islands (Fig. 1).

The Greater Antilles are among the most populated areas in the world, with the highest population densities of 411 km^−2^ in Puerto Rico and 377 km^−2^ in Haiti and the lowest of 101 km^−2^ in Cuba (Table 1). The per capita GDP ranges from greater than $25,000 in Puerto Rico, to less than $1,000 in Haiti. LCLUC differs greatly in the Greater Antilles. Jamaica, Puerto Rico, and Dominican Republic have evolved into the late reforestation stage with forest cover greater than 40%. Cuba is undergoing significant reforestation due to sugarcane shrinkage and thus has intermediate forest cover (almost 30%). Haiti is still in deforestation with low forest cover (FAOSTAT, faostat3.fao.org). The sharp contrast between high-level reforestation and extreme deforestation makes the Greater Antilles an ideal lab for studies of tropical forest transition and its consequences for carbon dynamics.

### Remotely sensed Vegetation Index and climate data

We chose MODIS datasets to quantify the greenness dynamics of the Greater Antilles in 2000–2015. MODIS sensors on board the Terra and the Aqua platforms provide reflectance information across wavelengths of 0.4–14.4 μm at the spatial resolution of 250 m–1 km (http://modis-land.gsfc.nasa.gov/). EVI, derived from the reflectance at blue, red, and near infrared bands, is proven to be more sensitive in high leaf biomass regime^32^ than the Normalized Difference Vegetation Index (NDVI), and therefore is more suitable for monitoring greenness in tropical forests. We used the level-3 MODIS Vegetation Index (VI) product (MOD13Q1, Collection 5) which provides EVI and pixel quality assurance (QA) for every 16 days at the spatial resolution of 250 m. MODIS EVI and QA values of the Greater Antilles in 2000–2015 (available from the middle February in 2000 to the end of July in 2015) were extracted using the Google Earth Engine platform (https://earthengine.google.com/). In particular, we first excluded pixels labelled as No Data in corresponding QA values and then filtered the remaining pixels accordingly to their QA values. Only pixels with QA values equaling 0 or 1 were kept for analysis (https://lpdaac.usgs.gov/dataset_discovery/modis/modis_products_table/mod13q1). For each country in Great Antilles, we summarized the spatial average and standard deviation of EVI for the pixels using Google Earth Engine, and derived the temporal trends of the two variables.

To explore the impacts of climate on greenness dynamics, we acquired the rainfall records at 20 meteorological stations available in Puerto Rico for the period of 2000–2015 (http://www.ncdc.noaa.gov/). Because of the limited temperature information from meteorological stations, we retrieved daytime and nighttime Land Surface Temperature (LST) for Puerto Rico from the MODIS LST products MOD11A2 and MYD11A2 with the spatial resolution of 1 km and temporal resolution of 8 days (http://modis.gsfc.nasa.gov/data/dataprod/mod11.php), as well as the corresponding Quality Assurance information. According to the description of QA values (https://lpdaac.usgs.gov/dataset_discovery/modis/modis_products_table/mod11a2), we only kept those pixels with the integer QA values as 0, 5, 17, and 21 to keep LST error less than 1 K.

To investigate the impact of cloud shading on changes in greenness, we also retrieved the daily daytime cloud information from the MOD35_L2 dataset (level 2, collection 6) at 1 km resolution (https://ladsweb.nascom.nasa.gov/api/v1/productPage/product=MOD35_L2). The categories of cloudiness of “confidently clear”, “probably clear”, “probably cloudiness”, and “confidently cloudiness” were converted into 0, 33, 66, and 100% of cloud cover, and were averaged over days to give average cloud cover for each year.

### Analyses of EVI trend for Great Antilles and its relationship with climate variables

To detect the difference in EVI among the five countries in Greater Antilles, we used spatial averages and standard deviations of EVI for each country at the time interval of 16 days. The spatial coefficient of variation (CV) was computed as the ratio of spatial standard deviation to the average and thus indicated the spatial heterogeneity of EVI. Time series regression with the first and second orders of autocorrelation was applied to derive the temporal trends by removing the seasonal variation. The magnitude (mean) and trend (slope) of EVI, its spatial standard deviation, and spatial coefficient of variation were compared among the five countries. To understand the trend of EVI, we analyzed land cover or forest cover information available for the Great Antilles from various global datasets, which include the global forest cover change in 2000–2012^15^ at 30 m resolution and the global forest area at country level provided by the World Bank (http://data.worldbank.org/indicator/AG.LND.FRST.ZS).

In order to investigate the climate impact on greenness, we intended to identify those areas without LCLUC based on land cover maps. Unfortunately accurate high resolution land cover maps are not available for most Caribbean countries except Puerto Rico^20^ for which a land cover map in 2000 is available at 30 m resolution^23^. Therefore, we identified vegetation patches without LCLUC in Puerto Rico for the study period based on the following information: (a) the land cover map of 2000, (b) aerial photographs with 0.4 m resolution in 2010 (www.gis.pr.gov), and (c) high-resolution images available at Google Earth after 2010. The selection criteria ensured that the sizes of the patches are large enough with internal homogeneous core areas and that the patches cover all vegetation types including pastures, drought deciduous woodlands, semi-deciduous and drought deciduous forests, seasonal evergreen and semi-deciduous forests, seasonal evergreen and evergreen forests, cloud forests, and wetlands.

**Figure 2:**
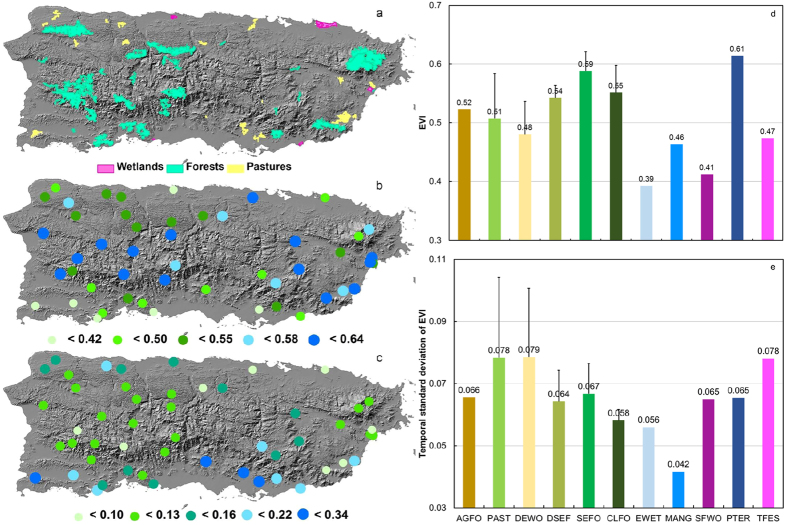
Spatial distribution of the vegetation patches without LCLUC (**a**), their mean EVI (**b**), and temporal coefficient of variation of EVI (**c**) in 2000–2015. Average EVI (**d**) and its temporal standard deviation (**e**) summarized for agricultural forest (AGFO), pasture (PAST), deciduous woodlands (DEWO), deciduous to seasonal evergreen forest (DSEF), seasonal and evergreen forest (SEFO), cloud forest (CLFO), emergent wetland (EWET), mangrove (MANG), seasonally flooded woodlands (SFWO), *Pterocarpus* freshwater swamp (PTER), and tidally flooded evergreen shrubland (TFES). Map created using ArcGIS 10.0 (Esri, CA, www.esri.com).

We started with selection of all continuous and homogeneous vegetation patches greater than 2,000 ha or the largest ones in their types from the land cover map of 2000. The patch sizes were reduced to their internal core areas by a buffer zone of 150 m in order to minimize the edge effects caused by microclimate and plant species composition/structure different from internal. The core areas were then checked against the aerial photos of 2010 and the recent high resolution images at Google Earth. The boundary of the patches was modified and patch area reduced when necessary to ensure the internal land cover without change. ArcGIS 10 (Esri, CA) was the main tool used in the procedure. A total of 51 patches without LCLUC were identified (Fig. 2a). In addition to reduced edge effects by taking core areas, using vegetation patch rather than pixel as the basic unit minimized the effects of spatial autocorrelations and lessened the impacts of missing pixel values when averaging EVI within each patch.

The time series of average EVI for each patch were extracted from MOD13Q1 accordingly by means of Google Earth Engine platform. We compared the temporal means and variations of EVI across vegetation types and topographic settings, i.e. windward facing northeasterly trade wind versus leeward. Time series analysis was applied to detect the trend of EVI for each patch.

The mean annual rainfall varies greatly in Puerto Rico from over 4,000 mm in the northeast cloud forest to less than 1,000 mm in the dry forest of the southwest. An increase of 100 mm annual rainfall could indicate less than 3% enhancement for the northeast but 10% rise for the southwest. We analyzed the rainfall trend of 20 meteorological stations across Puerto Rico in 2000–2015 to derive the temporal trend (slope) using time series analysis after missing values filled by means of Markov Chain to determine the probability of rain and Weibull distribution to determine the amount of rain. Relative rainfall trend defined as the temporal slope divided by the average rainfall of the station is used to promote the comparison of temporal changes while suppressing the sharp spatial rainfall gradient among the patches. Other than the annual rainfall amount, we also investigated the possible changes in rainfall pattern for stations with daily missing values less than 5%, which allowed us to identify intense rainfall event as daily rainfall greater than 95 percentile and dry days as daily rainfall less than 3 mm. We derived the temporal trends of number of annual dry days, number of consecutive dry periods, and the accumulated annual intensive rainfall and regular rainfall.

After filling the missing values, annual rainfall measured by the meteorological stations was Kriging interpolated at 30 m resolution over Puerto Rico. The interpolated annual rainfall was summarized for each vegetation patch to give time series of annual rainfall, from which mean annual rainfall and temporal trend of rainfall were derived for each vegetation patch without LCLUC.

Daytime and nighttime LST as well as daytime cloud cover were summarized for each vegetation patch. Time series of LST were available for 45, whereas cloud cover was available for all 51 vegetation patches. Means and temporal slopes of LST and cloud cover were derived in the way similar to the analyses of EVI and rainfall.

Finally, the trends (slopes) of EVI of the 45 vegetation patches were analyzed against the means and slopes of daytime and nighttime temperatures, rainfall, and cloud cover using partial correlation and multivariable regression. All statistics and time series analyses were done by means of the open access statistical software R^33^.

## Results

### Spatiotemporal variation of EVI across the Greater Antilles in 2000–2015

The mean EVI over space and time was significantly different among the five countries with the order of Jamaica > Puerto Rico > Dominican Republic > Cuba > Haiti (Table 2). We found that the average EVIs during 2000–2015 for all countries except Cuba were significantly decreasing, and the steepest decrease was found for Haiti. In contrast, the EVI of Cuba was increasing insignificantly. The comparison of temporal trend of average EVI reflected combined effects of climate changes and LCLUC on greenness. The analyses on the available land or forest cover change in the Caribbean were quite controversial. The global forest gain/loss dataset in 2000–2012^15^ indicated net loss for Dominican Republic, Haiti, Jamaica, and Puerto Rico, but net gain for Cuba (Supplementary Figure S1a). However, the country-level forest change reported by World Bank showed decreased forest cover for Haiti and Jamaica, but increased for Cuba, Dominican Republic, and Puerto Rico in 2000–2012 (Supplementary Figure S1b). The increase or decrease trends of forest change continued in 2000–2015 as reported by World Bank (Supplemental Table S2). The forest cover change in Cuba (positive), Haiti (negative), and Jamaica (negative) might contribute to the corresponding positive or negative EVI trends. However, the relationship between forest cover change and EVI trend in 2000–2015 among the 5 countries was not significant (*p* = 0.46, Supplemental Table S2).

Spatial coefficients of variation (CV) of EVI were significantly different among the countries, with the highest in Puerto Rico and Cuba and the lowest in Jamaica (Supplementary Table S1). The larger the spatial CV, the more heterogeneous the landscape is. Small spatial CV in Jamaica indicated its low spatial heterogeneity or high homogeneity of EVI. The spatial CV decreased during the studied period for all the counties except Jamaica, and the trend was significant for Puerto Rico, suggesting its landscapes as reflected in EVI were getting less heterogeneous or more homogeneous.

### Spatiotemporal patterns of EVI in vegetation patches without LCLUC in Puerto Rico

The average EVI across patches of woodland and forest was 0.55 ± 0.06 with the highest of 0.59 ± 0.03 for the seasonal evergreen and evergreen forests and the lowest of 0.48 ± 0.06 for the deciduous forests and woodlands (Fig. 2b and d). The average EVI across pasture patches was 0.51 ± 0.08. The average EVI for wetlands varied from 0.39 for emergent wetlands to 0.61 for *Pterocarpus* freshwater swamp. In general the average EVI was high in the mountains and the northeast facing the prevailing northeasterly trade wind, but relatively low in the leeward (southwest) of the central mountain (Fig. 2b). The temporal coefficient of variation in EVI exhibited a pattern almost opposite to that of average EVI: high in the leeward where most deciduous types reside, but much lower in the central mountains where most evergreen types locate (Fig. 2c).

**Figure 3:**
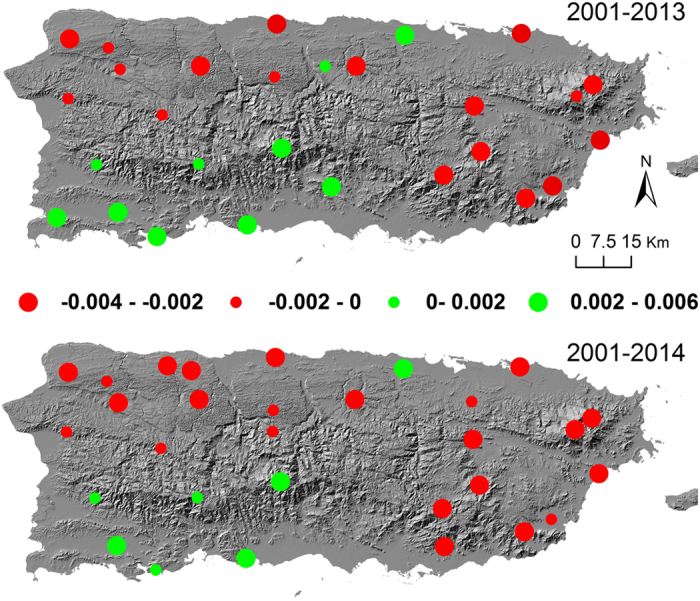
Temporal trends of EVI of the vegetation patches without LCLUC for the periods of January 1, 2001 – December 31, 2013 (upper) and January 1, 2001 – December 31, 2014 (lower). Green dots indicate significantly increasing trends while red ones significantly decreasing (*p* < 0.1). Map created using ArcGIS 10.0 (Esri, CA, www.esri.com).

Significant temporal trends of EVI (*p* < 0.1) in 2001–2013 were found for 3 cloud forest patches, 8 seasonal evergreen and evergreen forests, 3 seasonal evergreen and semi-deciduous forests, 3 deciduous woodlands/forests, 8 pastures, and 3 wetland patches (Fig. 3). Patches with negative temporal trend, implying decreasing EVI, were found mostly in the windward with great annual rainfall brought by the northeasterly trade wind. However, the increasing trend was found mostly for patches in the leeward under the rain shadow (Fig. 3). When a dry year of 2014 was added into the trend analysis, more patches with greater negative trend were found than those found for 2001–2013, but the spatial pattern of the trends remained the same.

### Spatiotemporal patterns of climate variables

**Figure 4:**
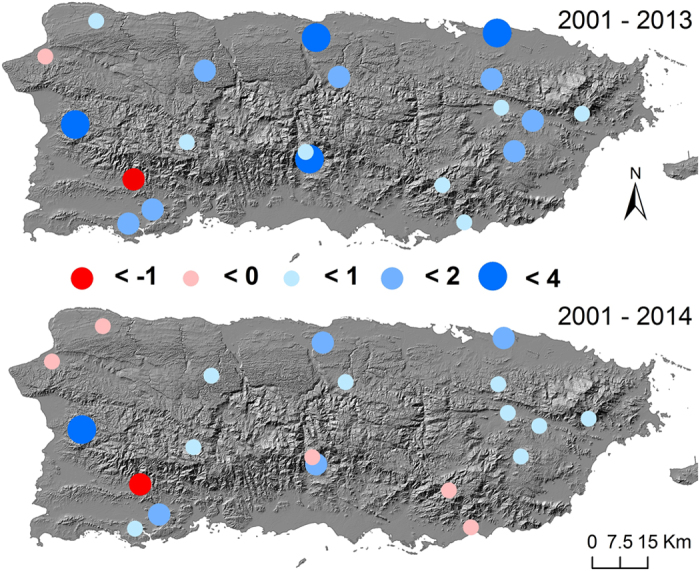
Relative rainfall trends for 20 weather stations in Puerto Rico in 2001–2013 (Upper) and in 2001–2014 (Lower). Blue and red dots stand for increasing and decreasing trends, respectively. Relative rainfall trend was calculated as the regression slope (mm yr^−1^) divided by the average annual rainfall (m) of each station. Map created using ArcGIS 10.0 (Esri, CA, www.esri.com).

The relative temporal rainfall trends of the 20 stations did not reflect the pattern of EVI trends between windward and leeward (Fig. 4 versus Fig. 3). The majority, 18 out of 20, weather stations showed increasing rainfall trends in 2001–2013, of which 7 were significant at *p* < 0.1 (Fig. 4 Upper panel). When the dry year of 2014 was added to the trend analysis, only 14 stations showed increasing rainfall with smaller slopes (Fig. 4 Lower panel) and only 3 out of 14 stations were significantly increasing.

For the 18 stations having increasing rainfall in 2001–2013, 17 stations showed decreased number of dry days, 13 decreased number of 7-day dry sequences, and 15 decreased number of 14-day dry sequences. Intensive rainfall increased for 15 stations at 19.5 ± 22.9 mm yr^−1^, whereas regular rainfall increased for 17 stations at 12.5 ± 10.2 mm yr^−1^.

The Kriging-interpolated annual rainfall for the vegetation patches during 2001–2014 echoed the results of the stations. The mean annual rainfall varied among the vegetation patches with the minimum, maximum, and mean as 966, 3,421, and 2,023 ± 537 mm, respectively. The slopes of rainfall for seven patches were negative, but for other patches were positive. The minimum, maximum, and mean slopes were −49, 32, and 6 ± 15.5 mm yr^−1^, respectively. However, none of the patches had significant rainfall trend.

**Figure 5:**
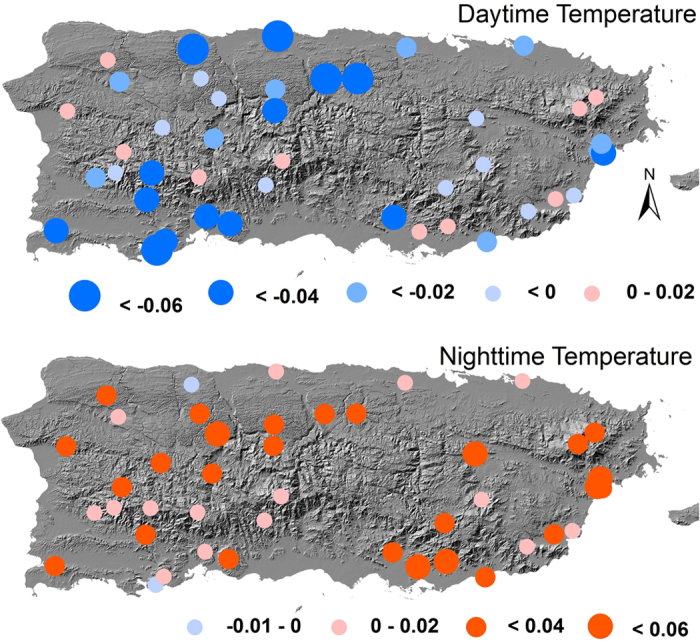
Annual trends (°C yr^−1^) in daytime (Upper) and nighttime (Lower) Temperatures in 2001–2014. Map created using ArcGIS 10.0 (Esri, CA, www.esri.com).

The temporal trends of daytime and nighttime temperatures were opposite to each other (Fig. 5). For 2001–2013, 41 out of 45 patches showed decreasing daytime temperature, of which 25 were significant at *p* < 0.05. The average trend in daytime temperature over all patches was −0.047 ± 0.039 °C yr^−1^. On the contrary, the nighttime temperature showed an average positive trend of 0.011 ± 0.015 °C yr^−1^. Thirty-four patches exhibited increasing nighttime temperature and the slope was significant for 10 patches at *p* < 0.05. For the period of 2001–2014, the average trend in daytime temperature was still negative at −0.027 ± 0.031 °C yr^−1^, but less negative than that for 2001–2013. Only 35 patches showed decreased daytime temperature, of which 20 were significant at *p* < 0.05 (Fig. 5 Upper panel). The nighttime temperature increased with greater magnitudes (0.022 ± 0.014 °C yr^−1^) in 2001–2014 than that in 2001–2013. Forty-three patches had increased nighttime temperature, and 26 of them were significant at *p* < 0.05 (Fig. 5 Lower panel).

The vegetation patches had the minimum, maximum, and mean daytime cloud cover of 0.31, 0.77, and 0.58 ± 0.11, respectively. The temporal trend (slope) of cloud cover over 2001–2014 varied from −0.0024 to 0.0036 yr^−1^, but none of the slopes had *p*-value lower than 0.15. The mean cloud cover trend was 0.00044 ± 0.0015 yr^−1^.

### Spatial pattern of EVI trend and its relationship with climate variables

**Figure 6:**
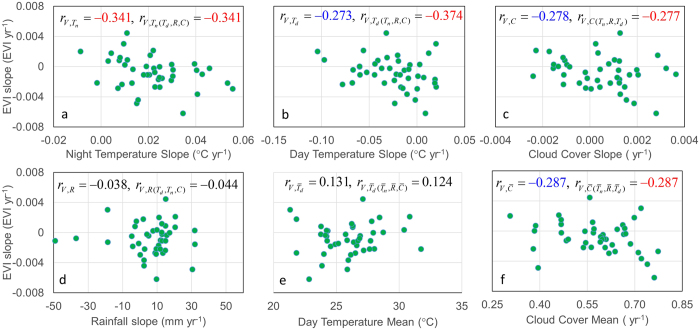
EVI trend (slope) vs. slopes of temperatures (**a** and **b**), cloud cover (**c**) and rainfall (**d**) and means of daytime temperature (**e**) and cloud cover (**f**). Correlation and partial correlation coefficients *r* represent the following variable key: *V, T_n_, T_d_, C*, and *R* stand for slopes of EVI, nighttime temperature, daytime temperature, cloud cover, and Rainfall, respectively, whereas variables with “^−^” denote the means. Subscripts within parenthesis are control variables used in the calculation of partial correlation. Correlation coefficients significant at *α* = 0.1 and 0.05 are light-blue and red colored, respectively.

Correlation and partial correlation between EVI slope and selected climate variables together with the scattered plots (Fig. 6) showed that EVI slope was only signficantly negatively correlated with the slope of nighttime temperature for α = 0.05 (Fig. 6a), but signficantly negatively correlated with the slopes of daytime temperature (Fig. 6b) and cloud cover (Fig. 6c) as well as mean cloud cover (Fig. 6f) for α = 0.1. However, all the partial correlation coefficients between EVI slope and these variables were significant for α = 0.05. EVI trend was not significantly correlated to the slope of rainfall (Fig. 6d) or mean daytime temperature (Fig. 6e).

The regression of the temporal slope of EVI during 2001–2014 on means and slopes of temperatures, rainfall, and cloud cover with stepwised selection of variables for minimum Akaike Information Criterion resulted in the following equation denoted by *V, C, T*_*d*_*, T*_*n*_, and *R* as the slopes of EVI, cloud cover, daytime temperature, nighttime temperature, and rainfall, respectively, and 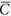, 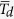, 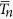 and 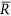 for the corresponding means.


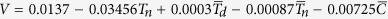


The regression had *p*-value as 0.02. Hence the temporal trend of EVI was reduced by the trend of nighttime temperature, the mean nighttime temperature, and the mean cloud cover, but promoted by the mean daytime temperature. The analysis of variance of the regression showed slope of nightime temperature and mean cloud cover explained 45% and 26%, and mean daytime and nighttime temperature accounted for 15% and 14%, of sum squares of regression, respectively. Therefore nighttime temerature rise and mean cloud cover dominated the impacts of climate variables on the temporal trend of EVI.

## Discussion

### Greenness trend and LCLUC in Caribbean

EVI dynamics is affected by both LCLUC and climate changes. The spatial greenness pattern in the Greater Antilles concurred well with the pattern of forest cover among the countries, i.e. the greater the overall greenness the higher the forest cover is (Table 2). We downloaded the global forest cover dataset^15^ and set forested area as those grid cells with forest cover greater than 30%^15^,^34^. The derived forest cover in 2000 among the five countries in the Caribbean has a correlation of 0.97 with the corresponding average EVI (Supplemental Table S3). Four out of five countries showed significantly decreasing trends of EVI in 2000–2015, while EVI in Cuba was increasing partly due to the strong reforestation (Supplemental Figure S1b) reported recently^15^,^20^. Based on the assumption of similar climate tends in the Great Antilles, the significant reduction of difference in EVI between Cuba and other countries with high forest cover highlighted the faster reforestation/afforestation in Cuba (Table 2). Haiti had the steepest decreasing trend of EVI because of continuous deforestation for charcoals. The continued reforestation in Puerto Rico (Supplemental Figure S1c) makes the greenness more homogeneous as reflected in the significantly decreasing spatial CV of EVI (Supplementary Table S1). Although there is strong correlation between EVI and forest cover, the temporal trend of EVI is not explained by changes in forest cover (*p* = 0.46, Supplemental Table S2). The directional change of forest cover in 2000–2012 is in consensus for Cuba, Jamaica, and Haiti between the global forest dataset by Hansen *et al*.^15^ and the country-level forest area by World Bank (Supplemental Table S2b), but contradictory for Puerto Rico and Dominican Republic. The National Land Cover Database (http://www.mrlc.gov) in 2001 and 2011 and the local LCLUC study in 2000 and 2010 (unpublished data) both supported the increased forest cover reported by World Bank (Supplemental Figure S1c). The contradictory negative trend retrieved from the global forest dataset^15^ might be attributed to difference in forest definition and spatial resolution, and overestimation of forest cover and forest loss in tropical region as reported by recent studies in Indonesia and South America^34^,^35^. These controversial reports also remarked that great care should be taken to apply global LCLU datasets to regional and local investigations.

### Explanation of spatially heterogeneous greenness trend for vegetation patches

The analysis of vegetation patches without LCLUC revealed distinct EVI values, seasonal dynamics, and interannual trends among the vegetation types. Forest patches in general have higher EVI than pastures. The seasonal evergreen and evergreen forests have higher EVI than dwarf cloud forests. The deciduous forests show the lowest overall EVI due to massive leaf fall in dry seasons (Fig. 2). The temporal variation of EVI, indexed by the temporal coefficients of variation, mostly describes the seasonality of vegetation greenness, which is much higher in the leeward with dry deciduous forest than in the mountains (Fig. 2c).

Surprisingly, the temporal trend of EVI varies greatly with significant increase of greenness in the rain shadow leeward but significant decrease in the windward with great rainfall interception (Fig. 3). Considering the spatial distribution of vegetation types, EVI of the moist forest in the north and east has decreased but that of the dry forest found mostly in the southwest has increased. Recent IPCC assessment report^5^ highlighted that temporal trend is sensitive to the selected time period. To reduce the artificial effect of incomplete yearly data in 2000 (starting in the middle of February) and 2015 (ending in the end of July), we chose the years with complete data for trend analysis, e.g. Jan. 2001 – Dec. 2013 (Fig. 3 Upper) and Jan. 2001 – Dec. 2014 (Fig. 3 Lower). The drought in 2014 brought down the EVI trend in both windward (more negative) and leeward sides (less positive), but the pattern of negative in the windward and positive in the leeward remained.

Our correlation and regression analyses conclude that night warming is the primary factor that discerns the spatial pattern of EVI trend. Excessive night warming is thus the major driver for the decreasing trend of greenness in Puerto Rico despite the increasing forest cover. Nighttime warming in the tropics was believed to determine interannual variation of productivity at local scale^36^ and to dominate variations in global terrestrial C sink^10^. Warming in night elevates the daily minimum temperature, raises the baseline maintenance respiration, offsets the daytime carbon gain, and reduces the amount of carbon for leaf growth. Decreases in greenness will occur when the leaf growth is exceeded by the leaf senescence. Spatial pattern of nighttime warming explains the topographic pattern of greenness trend (Fig. 3). The average nighttime warming of the leeward patches in 2001–2014 (Green dots in southwest, Fig. 3) ranges from **−**0.0086 (nighttime cooling) to 0.0096 °C yr^−1^, much lower than the island average of 0.022 ± 0.014 °C yr^−1^ (Fig. 5).

The spatial pattern of night warming may be a result of the heterogeneous distribution of clouds (Supplemental Figure S2): The heavier cloud cover in the windward tends to block more longwave radiation from land surface during the night than the lighter cloud in the rain shadow leeward does, resulting in stronger night warming in the windward. Our result indicates daytime cloud as the second important factor contributed to strengthen the difference in EVI trend among the vegetation patches. Dense cloud in the windward also tends to block more shortwave radiation thus to allow less photosynthetic photon flux density to reach the canopy than light cloud in the leeward does. Radiation is the major limiting factor for plant growth in the warm and wet/moist tropics^37^. Great amount of rainfall in tropics usually associates with frequent cloudy days, which tends to reduce shortwave radiation and primary production^13^,^29^,^37^.

The insignificantly increasing rainfall and cloud cover, as well as the cooling daytime temperature, did not contribute significantly to explain the spatial pattern of greenness trend in the regression model despite their significant partial correlations with EVI trend (Fig. 6b and c). Daytime cooling as a result of increased rainfall and cloud seems to favor the positive trend of greenness (Fig. 6b) by enhancing carbon assimilation, reducing daytime maintenance, and alleviating drought for the warm tropics. However, what appeared in the regression model is the positive effect of the mean daytime temperature, showing greater mean daytime temperature is associated with stronger positive, but weaker negative trend of greenness. Mountains with higher elevation and lower temperatures have less fine soil and poorer nutrient balance due to strong erosion but weak mineralization and weathering than the lowland with higher temperature. Hence the greenness trend in the lowland tends to be greater than the upper land. We also found a strong and significant correlations between mean daytime temperature and daytime cooling (*r* = 0.485) so that the higher the mean daytime temperature the stronger the daytime cooling.

Although there are many studies highlighting the impact of asymmetric effects of nighttime versus daytime temperature on vegetation growth, most of them deal with warming in both day and night^10^,^11^,^30^,^38^,^39^, and few studies are found for tropical forests^40^. Our study revealed a case in tropical vegetation with simultaneous nighttime warming and daytime cooling. The synergetic effect of daytime cooling and nighttime warming resulted in overall reduced EVI in general in spite of mild increase in rainfall. Nighttime warming was negatively linked to greenness in this study and others^10^,^11^,^36^. However, it was also reported to enhance greenness via overcompensation of photosynthesis in grassland^11^,^41^ or alleviation of frost damage in Tibet Plateau^30^. A global analysis of NDVI trend also reported a positive relationship with average temperature^42^. Future studies to consider various drivers, such as climate, LCLUC, CO_2_ fertilization, and nutrients, are needed to build comprehensive understanding of the mechanisms behind.

The relationship between rainfall and vegetation growth is nonlinear^4^. In general, vegetation growth increases with enhanced rainfall, but after a threshold at about 3,000 mm, vegetation grows better under less rainfall due to enhanced soil oxygen content and less leaching loss of nutrients. However, changes in cloud cover and shortwave radiation would complicate the rainfall effect depending on energy-limited or water-limited. The long-term onsite studies at representative forests, such as LUQ-LTER for cloud forest and moist forest and Guanica-NEON for dry forest, would help reveal the nonlinear mechanisms in plant physiology and biogeochemical cycles in response to climate changes. The study in Puerto Rico and Caribbean region has the potential to be scaled up to regional interactions between climate and vegetation. These studies are urgent for improving Earth system models given the facts of the global impact of tropical nighttime warming on terrestrial C sink^10^, the faster warming at night than daytime^5^, the largest plant C pool and NPP of tropical forest, and the relatively low soil C residence time^4^.

## Additional Information

**How to cite this article**: Yu, M. *et al*. Extent of Night Warming and Spatially Heterogeneous Cloudiness Differentiate Temporal Trend of Greenness in Mountainous Tropics in the New Century. *Sci. Rep.* **7**, 41256; doi: 10.1038/srep41256 (2017).

**Publisher's note:** Springer Nature remains neutral with regard to jurisdictional claims in published maps and institutional affiliations.

## Supplementary Material

Supplementary Figure

## Figures and Tables

**Table 1:** Natural and socioeconomic information of the Great Antilles (Data source: The World Bank, data.worldbank.org; FAOSTAT, faostat3.fao.org).

Country	Area (km^2^)	Population per km^2^	GDP per Cap	Dominant Vegetation	LCLUC Feature
Puerto Rico	8,950	411	>$25 k	Moist forest	Reforestation, demand for agriculture
Cuba	110,860	101	~$5 k	Dry forest, wetland	Reforestation, Sugarcane abandonment
Dominican Republic	48,730	216	~$5 k	Moist forest	Reforestation
Jamaica	10,990	255	~$5 k	Moist forest	Stable
Haiti	27,750	377	<$1 k	Moist forest	Deforestation for energy

**Table 2:** Trends of EVI in the Greater Antilles in 2000–2015.

Countries	Intercept	Annual Slope
PUE	0.48	−0.00095**
JAM	0.50	−0.00096**
DOM	0.44	−0.00089*
CUB	0.41	0.00032
HAI	0.40	−0.00110**
PUE-JAM	−0.017**	0.00005
PUE-DOM	0.038**	−0.00008
PUE-CUB	0.063**	−0.00135*
PUE-HAI	0.088**	0.00014
JAM-DOM	0.055**	−0.00013
JAM-CUB	0.081**	−0.00134**
JAM-HAI	0.105**	0.00009
DOM-CUB	0.026**	−0.00121*
DOM-HAI	0.050**	0.00023
CUB-HAI	0.024**	0.00144**

PUE, Puerto Rico; JAM, Jamaica; DOM, Dominica Republic; CUB, Cuba; HAI, Haiti. **Significant at 0.05, and *significant at 0.1.
