# Supplementary material for: Extent of Night Warming and Spatially Heterogeneous Cloudiness Differentiate Temporal Trend of Greenness in Mountainous Tropics in the New Century
Source: Sci Rep. 2017 Jan 25;7:41256. doi: 10.1038/srep41256 (PMC5264156; doi:10.1038/srep41256)
Supplement: Supplementary Figure [file srep41256-s1.pdf]

Extent of Night Warming and Spatially Heterogeneous Cloudiness Differentiate Temporal Trend of Greenness in Mountainous Tropics in the New Century

Mei Yu*[1], Qiong Gao[1], Chunxiao Gao[2], and Chao Wang[1]

[1]University of Puerto Rico, Rio Piedras, San Juan, PR 00936, USA; [2]Duke University, Durham, NC 27705, USA * Email: meiyu@ites.upr.edu; Tel: 1-787-764-0000 x 4375

Supplementary Table S1

Table S1 Trends of Spatial CV of EVI in the Greater Antilles in 2000 – 2015. PUE, Puerto Rico; JAM, Jamaica, DOM, Dominica Republic; CUB, Cuba; HAI, Haiti, **, significant at 0.05, and *, significant at 0.1.

Countries	Intercept	Annual Slope
PUE	0.37	**-0.00063***
JAM	0.31	0.00019
DOM	0.34	-0.00006
CUB	0.37	-0.00027
HAI	0.33	-0.00007
PUE-JAM	0.05**	**-0.000824****
PUE-DOM	0.02**	-0.000327
PUE-CUB	-0.01**	-0.000257
PUE-HAI	0.03**	**-0.000514***
JAM-DOM	-0.03**	0.000418
JAM-CUB	-0.06**	0.000491
JAM-HAI	-0.02**	0.000291
DOM-CUB	-0.03**	0.000259
DOM-HAI	0.01**	-0.000164
CUB-HAI	0.04**	-0.000246

Supplementary Table S2

Table S2 Forest Change summarized from Hansen et al. (2013) and World Bank, and EVI trend in the Greater Antilles. PUE, Puerto Rico; JAM, Jamaica, DOM, Dominica Republic; CUB, Cuba; HAI, Haiti.

Countries	Forest Cover Change in 2000 – 2012 (%, Hansen et al. 2013)	Forest Cover Change in 2000 – 2012 (%, World Bank)	Forest Cover Change in 2000 – 2015 (%, World Bank)	EVI trend in 2000 – 2015 (per year)
PUE	-0.86	4.05	5.17	**-0.00095****
JAM	-2.37	-0.42	-0.53	**-0.00096****
DOM	-3.16	8.23	10.29	**-0.00089***
CUB	0.49	5.88	7.39	0.00032
HAI	-0.84	-0.35	-0.44	**-0.00110****

Supplementary Table S3

Table S3 Forest Cover in 2000 retrieved from the global forest dataset (Hansen et al. 2013) by setting forested area as those pixels with forest cover greater than 30%, and average EVI in 2000 in the Greater Antilles. PUE, Puerto Rico; JAM, Jamaica, DOM, Dominica Republic; CUB, Cuba; HAI, Haiti. The correlation coefficient is 0.97 (p = 0.006).

Countries	Forest Cover in 2000 (%)	EVI in 2000
PUE	58.17	0.47
JAM	70.08	0.49
DOM	53.19	0.43
CUB	36.39	0.40
HAI	31.17	0.38

Extent of Night Warming and Spatially Heterogeneous Cloudiness Differentiate Temporal Trend of Greenness in Mountainous Tropics in the New Century

Mei Yu*[1], Qiong Gao[1], Chunxiao Gao[2], and Chao Wang[1]

[1]University of Puerto Rico, Rio Piedras, San Juan, PR 00936, USA; [2]Duke University, Durham, NC 27705, USA

* Email: meiyu@ites.upr.edu; Tel: 1-787-764-0000 x 4375

Supplementary Figure S1

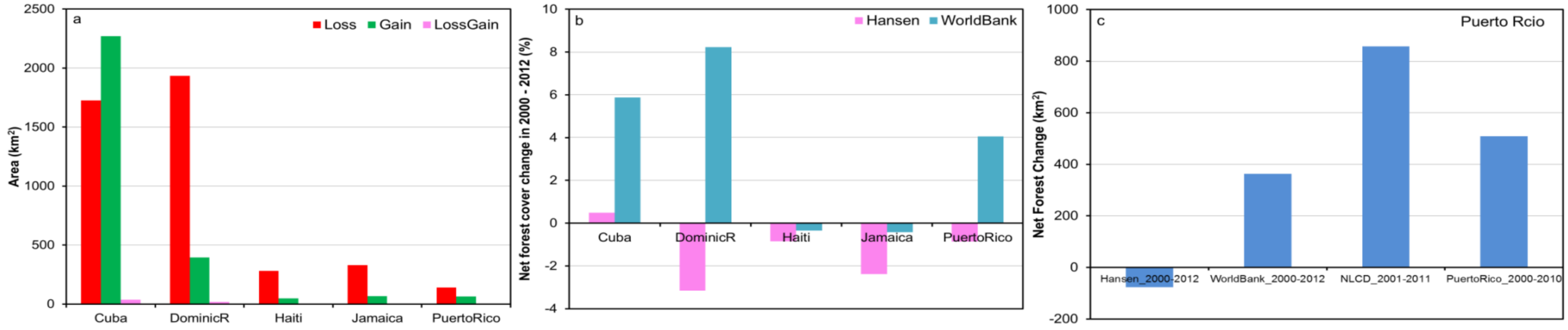

Figure S1 Forest Changes in the Great Antilles during the first decade of 21st century. a) Forest Gain / Loss in 2000 – 2012 from a global forest cover change dataset (Hansen et al. 2013, at 30 m resolution). b) Net forest cover change in 2000 – 2012 from the global forest cover change dataset (Hansen et al. 2013, at 30 m resolution) and the global forest cover dataset (World Bank, at country level, http://data.worldbank.org/indicator/AG.LND.FRST.ZS). c) Net Forest Change in Puerto Rico from 4 data sources at local, national, and global scales. Global forest cover change dataset in 2000 – 2012 (Hansen et al. 2013), Global forest cover dataset in 2000 – 2012 (World Bank), National Land Cover dataset in 2001 – 2011 (at 30 m resolution, http://www.mrlc.gov/nlcd01_data.php), and Puerto Rico land cover mapping in 2000 – 2010 (at 30 m resolution, manuscript).

Supplementary Figure S2

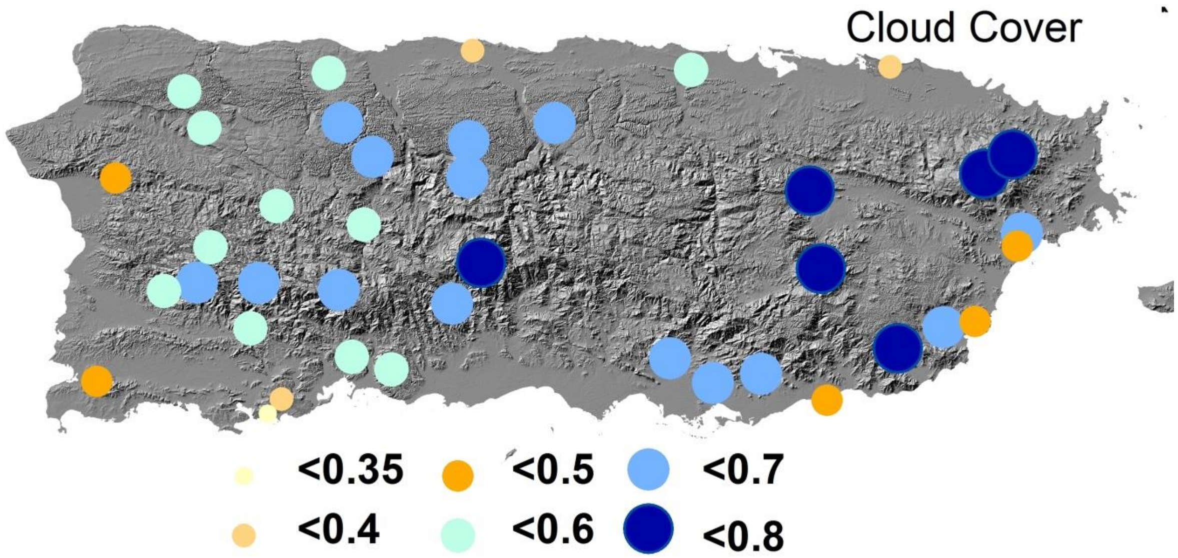

Figure S2 Average cloud cover in 2001 – 2014 in Puerto Rico. Map created using ArcGIS 10.0 (Esri, CA, www.esri.com).
